# Azoximer Bromide: Mystery, Serendipity, and Promise

**DOI:** 10.3389/fonc.2021.699546

**Published:** 2021-09-10

**Authors:** Lyudmila Yuryevna Grivtsova, Natalia Alexandrovna Falaleeva, Nikolay Nikolaevich Tupitsyn

**Affiliations:** ^1^A. Tsyb Medical Radiological Research Centre, National Medical Research Radiological Centre of Ministry of Health of the Russian Federation, Moscow, Russia; ^2^Laboratory of Immunology of Hematopoiesis, N.N. Blokhin Cancer Research Center (RCRC), Moscow, Russia

**Keywords:** oncology, immunology, infectious disease, serendipity, azoximer bromide, immunomodulator

## Abstract

Azoximer bromide (AZB) was identified as an immunomodulator, and was initially developed and currently successfully indicated as one of several natural polyelectrolytes, a vaccine adjuvant, and an effective agent for the treatment of infectious and inflammatory diseases of viral, bacterial, and fungal origin. AZB has the potential to increase an individual’s resistance to local and general infection and is indicated for the treatment of viral infections, and has also demonstrated clinical efficacy in the treatment of a variety of secondary immunodeficiencies. However, AZB may offer long-term promise beyond use against infection. Multiple clinical trials and research studies in cancer patients have reported favourable outcomes with AZB as well as an optimal safety and tolerability profile. The findings raise the possibility of direct antitumor properties. This literature review analyses the novel mechanisms that mediate the AZB direct anticancer effects. Overall, the evidence suggests that AZB has the hallmark of an agent that could be used to support existing cancer treatments at different stages of disease.

## Introduction

The early identification and success of immunomodulatory molecules known as adjuvants (from the Latin word adiuvare meaning ‘to help’) in enhancing immune responses to antigen-based vaccines, could at best be called empirical in fashion (1, 2). Alums (aluminium, phosphate, or hydroxide) were the first molecules to be used as adjuvants and were introduced in the 1920s for vaccines against diphtheria and tetanus toxoids. They have since been employed successfully to formulate most of the non-living vaccines that have been administered to billions of infants and adults (3, 4).

Unknown to many, research at the State Scientific Centre of the Institute of Immunology of the Ministry of Health of the Russian Federation (now the FSBI State Scientific Centre ‘Institute of immunology’ FMBA of Russia) during the 1970s and 80s was focused on identifying adjuvants for flu vaccines, aimed at enhancing the immune responses to infection (5–7). At the same time of the release of the highly successful oil-in-water squalene-based immunologic adjuvant MF59, the Russian team registered and commercialised azoximer bromide (AZB; Polyoxidonium^®^). AZB was the lead compound derived from a class of heterochain aliphatic polyamines and it was licenced along with a hemagglutinin glycoprotein-based influenza vaccine, Grippol^®^ (8). Move forward to the present day and variants of the adjuvant-glycoprotein combination have been widely used in Russia and Slovakia since the early 2000s and are also currently available in Georgia, Belarus, Ukraine, Kazakhstan, and Uzbekistan. Estimates suggest that the adjuvant AZB has been used in over 400 million doses of influenza vaccine (9, 10).

A Russian Federation patent to support the clinical use of polymeric compounds with immune-stimulating activity was awarded in 1996 and the team responsible for the development of AZB established the first local commercial pharmaceutical company (Petrovax NPO) under the leadership of Professor Arkady Nekrasov (11). The question remains, is AZB a safe and effective medicine that has been widely overlooked in the West or is the available scientific and clinical evidence insufficient to promote its widespread adoption? Preliminary work leading up to the discovery of AZB was published in the 1970’s and 80’s but at the time it was generally accepted that research from the Russian institutions would publish in the Russian language in Russian scientific journals. This was during the early days of electronic databases, coverage was sketchy and Index Medicus remained the bibliographic database of life science and biomedical science information up until 2004 (12). The titles of articles not published in English would be translated but professional translations would be required if you could get a copy of original articles (13). Most likely this part explains why the work with AZB went unnoticed. Over the last two decades researchers in the former Soviet block countries continued to publish their work on AZB. However, most of these publications continue to be in Russian language journals (14). It is possible that isolation during the years of the Cold War saw divergence in the way science is reported and Russian scientists have found it challenging to adjust to the strict requirements of modern publishing houses (15, 16). Although modern research engines and databases can provide ready translations (of research abstracts at least) there is a certain bias against research published in Russian language. English remains the lingua franca of the biomedical sciences and access to full translations of research papers remains a challenge and, unlike the majority of international journals, most articles in Russian journals are hard to search and sources cannot be captured by citation management systems (they do not have special coding of articles descriptors) (17). Many Western research scientists continue to view research from Russia to be poor. This is not helped by too many publications being seen to be of poor quality, reporting inappropriately designed studies and incorrect statistical analyses (17, 18). This seems to leave the global scientific community with little appetite to engage with such publications or collaborate with Russian scientists and physicians (19). The issue of mistrust has been identified and gradual implementation of common international standards to better fit with guidelines such as those issued by ICJME, have been initiated (14).

From the outset, AZB was identified as an immune modulator that had the potential to increase a host’s resistance to local and general infections. AZB was initially developed and currently successfully indicated as an effective agent for the treatment of infectious and inflammatory diseases of viral, bacterial, and fungal origin (20). The mechanism of action of AZB on numerous components of the immune system has been studied in detail since 1983 (21). It was established that AZB influences the activation of innate immunity, phagocytosis, humoral and cellular immunity. Local (nasal, sublingual) use of AZB activates early defences against infection, including the bactericidal properties of neutrophils and macrophages, enhances phagocytosis of bacteria, and also increases the bactericidal properties of saliva and mucous membrane secretion in the upper respiratory tract (22). The ability to block soluble toxic substances and microparticles is another feature of AZB, which enhances the removal of heavy metal salts from the body, as well as inhibiting lipid peroxidation, both through the capture of free radicals and through the elimination of catalytically active Fe^2+^ ions (23). In addition, AZB has demonstrated clinical efficacy in the treatment of a variety of secondary immunodeficiencies, such as chronic, recurrent, or indolent infectious and inflammatory diseases (skin and soft tissues, eyes, bronchopulmonary apparatus, gastrointestinal, urogenital), as well as, acute bacterial and viral infections (20).

The immunomodulatory action of AZB is based on both activation of phagocytic cells and natural killer cells as well as stimulation of antibody formation and synthesis of both interferon-alpha (IFN-α) and interferon-gamma (IFN-γ) (24). It activates the three most important phagocytic subpopulations, including motile tissue macrophages, circulating blood phagocytes, and resident reticuloendothelial phagocytes. In addition, AZB activates macrophage migration, phagocytosis, and digestion of pathogenic bacteria, encouraging the capture and removal of foreign microparticles from circulating blood. It is also associated with increased antibody production in response to various antigen types by increasing the efficiency of the interaction between T- and B lymphocytes (24, 25). In the absence of antigenic stimulus, AZB does not induce any polyclonal transformation of B-lymphocytes and does not cause multiple cycles of T- and B-lymphocyte cell division, distinguishing it from bacterial mitogens.

AZB also demonstrates antitoxic properties, protecting cell membranes against cytotoxic action, and reducing the toxicity of pharmacological drugs. It penetrates the cell endosomal compartment, where it is associated with increased micromolecular concentrations of hydrogen peroxide (H_2_O_2_), an activator for various signalling molecules and transcription factors, in particular nuclear factor kappa B (NF-κB) (25, 26). It also has detoxifying and antioxidant properties which are largely determined by the drug’s structure and high molecular weight. *In vitro* studies demonstrated multiple effects of AZB, including an increase in degranulation of natural killer cells, an increase in T cell proliferation, and the expansion and maturation of dendritic cells with the expression of several co-stimulatory molecules. In addition, the administration of AZB in experimental animals with inoculated tumours has led to a decrease in tumour growth (26, 27). More recently, it has been demonstrated that common signalling pathways play a role in both antiviral and anticancer responses (28–31).

## From Research Tool to Medicine

During its development, AZB was considered a research tool, one of several natural polyelectrolytes (polysaccharides, native nucleic acids, double-strand synthetic polynucleotides) that were known to activate the immune system toward other antigens, that is, serve as immunomodulator (4, 8, 32). As such, AZB was employed in a broad range of *in vivo* and *in vitro* investigations, looking at the development of linear synthetic polyelectrolytes with diverse structures (mainly structural analogues of non-antigenic biopolymers unknown to the immune system). Serendipitously it was noticed that AZB intensified the formation, migration, and dissemination of precursor stem cells, and functional immune cells (26). Stedman’s Medical Dictionary ‘serendipity’ refers to ‘an accidental discovery;’ i.e., ‘finding one thing while looking for something else’ (33). Serendipity is one of the many factors that have contributed and continue to contribute to the discovery of modern treatments, including AZB (34, 35). The English scholar, Horace Walpole (1717–1797), first coined the word ‘serendipity’ in 1754, and today, the word has come to represent “the faculty of making happy and unexpected discoveries” (35).

When synthetic polyelectrolytes including AZB were incubated with typical antigens (proteins, natural microbial polysaccharides, and their synthetic analogues) they appeared to serve as immunomodulators, augmenting responses (26, 36). Moreover, individual bacterial or viral antigens, not sufficiently active by themselves, induced specific immune responses, enhanced by several orders of magnitude if chemically bound to synthetic polyelectrolytes (37). It was these characteristics that resulted in commercialisation of AZB as both a vaccine adjuvant and an immunomodulatory agent.

Over the last 20 years AZB has been used extensively with few safety concerns being reported during clinical development and routine post-authorisation, pharmacovigilance (10). Research with AZB has continued and a considerable number of clinical trials/research studies with AZB have provided data on exposure in more than 5,000 subjects. These clinical data indicate that AZB is effective across a range of conditions, including bronchial asthma (38–41), chronic recurrent herpes simplex infections (42), pneumonia (43), pyelonephritis (44), and recurrent urogenital chlamydial infections (45, 46). Where side effects have been reported they have included restlessness, fatigue, feeling hot/pyrexia, and asthenia (10). The tolerability profile has seen AZB become an agent of choice for many disease conditions associated with abnormalities of the immune system across the Russian Federation and Slovakia as well as Georgia, Belarus, Ukraine, Kazakhstan and Uzbekistan. Use includes chronic bronchitis, recurrent furunculosis, generalised forms of surgical infections, purulent-septic infections of the lungs and pleura, atopic diseases (i.e., obstructive bronchitis, atopic dermatitis with signs of immunological abnormalities), purulent-inflammatory ENT diseases, persistent chlamydial urogenital infection, pulmonary tuberculosis, acute urticaria, chronic inflammatory diseases, as well as, viral and bacterial infections (47). It should however be noted that in most cases, AZB leads to better outcomes when added to ‘standard of care’ therapies, and studies have rarely included controlled comparisons.

The recorded side effect and tolerability profile of AZB in combination with a broad range of influenza virus antigens (involving about 50 million recipients in the Russian Federation), confirms its safety and feasibility for use in commercial vaccines (9, 10). Serendipity surfaced once again early in 2020. Research findings quickly identified increasing reports of severe acute respiratory syndrome, COVID-19, being due to a new virus, severe acute respiratory coronavirus 2 (SARS-CoV-2). As the 2020 pandemic manifested, physicians characterised a spectrum of clinical presentations ranging from asymptomatic infections to life-threatening illness and mortality (42). More severe disease was characterised by infection of the lower respiratory tract, pneumonia, and respiratory failure, which results in death in about 0.5% of confirmed cases (43). In Russia, more than a million cases of SARS-CoV-2 infection were registered in the first 6 months, with over a million recovering and more than 23,000 recorded deaths (44). Although immunotherapies and vaccines were promised to be delivered in Russia, they were slow in arriving, and there remains no confirmed effective treatments (45, 46). Percutaneous tracheostomy under endoscopic control was generally adopted as standard practice. In addition to minimising complications associated with an endotracheal tube versus surgical tracheostomy, tracheostomy was believed to have a lower potential for aerosol formation and thus posed a lower risk of viral exposure load on medical staff (23). Nevertheless, any procedure undertaken in the care of patients carried the risk of aerosol formation and the exposure of healthcare workers to infection. Russian physicians felt that they had access to a pharmacological defence in the form of AZB, which was believed to increase an individual’s resistance to local and general infection and indicated for the treatment of viral infections (48).

Initially, reports started to emerge on the prophylactic use of AZB in physicians and healthcare teams working directly with patients, and later in observational studies conducted on patients presenting at hospital (49–51). Although not made as part of formal studies, these observations indicated possible benefits of AZB in COVID-19 that warranted further investigation. An appropriately powered, a well-controlled study was designed and is currently underway (49).

## Clinical Promise Beyond Infection

It is generally assumed that the current COVID-19 crisis will pass as the infection burns itself out, a level of herd immunity is established across the global population, or through vaccination bringing the current pandemic under control. It is hard to know what role AZB may play in any of these future scenarios, either as an aetiotropic therapy or as an adjuvant in future vaccines. However, AZB does offer long-term promise beyond infection.

Around the same time that AZB was being used in its first patients, thalidomide was approved by the United States (US) Food and Drug Administration (FDA) for use in the US for the treatment of erythema nodosum leprosum, an inflammatory condition associated with leprosy. Subsequently, and based on pioneering investigations at the University of Arkansas, thalidomide alone or in combination with dexamethasone was demonstrated to be effective in the treatment of multiple myeloma. The development of immunomodulatory drugs (IMiDs) swiftly followed these discoveries, seeing novel second generation agents expressing major anti-myeloma activity and more manageable toxicity profiles. These agents have since been shown to be highly successful in the treatment of a variety of conditions and have heralded a new era of anti-cancer therapy, engaging our immune system and offering hope to a generation of people suffering from cancer (52).

Interestingly, early studies showed that AZB has immunomodulatory properties raising the possibility that it may have direct antitumor properties, though perhaps acting *via* a different mechanism of action from the IMiDs. One candidate pathway is the melanoma differentiation-associated protein 5 (MDA5) gene, which acts as a modifier of the innate immune response, however, this seeks further investigation (17, 22). AZB also inhibits the accumulation of the myeloid-derived suppressor cells (MDSCs) in conditions of experimental aseptic inflammation as well as reducing extracellular neutrophil trap formation in *in vitro* studies (53, 54). It enhances the expression of the inducible costimulatory-ligand (ICOS-L) molecule by 1.7-fold and increases the ability of dendritic cells to stimulate the maturation of follicular helper T-lymphocytes and enhances the T-dependent humoral response (55, 56). In short, AZB displays properties of a drug that might be expected to have direct antitumor effects.

*In vitro*, AZB binds to human peripheral blood monocytes and neutrophils, and a lesser extent to lymphocytes, more over the drug product also has immunogenic properties. These include stimulation of IL-6 production, increasing bactericidal activity of white blood cells, activating the production of H2O2, and improving neutrophil and macrophage phagocytosis (24, 25, 57). Moreover, AZB contributed to a significant expansion of CD4^+^ T-cells *in vitro* with a possible role in mediating both, humoral and cell-mediated immunity by activating the cytotoxic response of lymphocytes *via* dendritic cells (26). A recent study looking at the expression of toll-like receptor genes (TLR 2, 3, 4, 7, 8, 9) and two cytosolic gene receptors of viral nucleic acids (RIG-I and MDA5) on the acute monocytic leukaemia cell line (THP-1), found that presence of AZB activated TLR 4 and resulted in multiple activations of the MDA5 gene transcription (58). Another property of AZB is its ability to reduce the level of NET formation in experimental studies *in vitro* (45). Moreover, it was shown that AZB leads to the secretion of cytokines by T-helper 2 (TH-2) cells, including interleukin-5 (IL-5) (27, 55, 56). AZB has a complex effect on the immune system. This results in a significant increase in expression of ICOS-L molecule by 1.7-fold, thus increasing the ability of dendritic cells to stimulate the maturation of T-follicular CD4^+^ cells and enhance humoral response that may contribute to the mechanism of antibody-dependent cellular cytotoxicity. Its effect on the cytosolic protein MD5 leads to the activation of tumour cell apoptosis *via* innate immune cells. In addition, the drug product increases phagocytosis and reduces the ability of neutrophils to form extracellular neutrophil traps. Thus, AZB appears to show all the signs of an agent that could support existing therapeutic regimens at different treatment stages in cancer patients.

AZB has been investigated in three established *in vivo* oncological models: Lewis lung carcinoma, pleural mesothelioma, and spontaneous carcinogenesis. During these experimental studies, an anticancer effect of AZB was observed, but it was less pronounced in the more aggressive tumour (Lewis lung carcinoma). More specifically, AZB significantly delayed the growth of Lewis Lung carcinoma in mice, by 32.5% in accordance with the tumour mass. Two weeks after AZB administration, only 10% of mice died in the experimental group and 30% in the control group (59). For tumours with lower malignancy (inoculated rat pleural mesothelioma, spontaneous haemoblastosis in mice), more apparent positive results were obtained. It should be noted that AZB administration in the presence of inoculated tumour has led in no case to increased neoplastic process.

Initial clinical studies with AZB aimed to augment existing therapies by improving tolerance of chemotherapy and major surgical interventions, as well as, patients’ quality of life (**Table 1**). In a double-blind placebo-controlled study of AZB as an adjuvant with 5-fluorouracil (stage III-IV colon cancer) in patients undergoing radical or palliative surgical treatment, exposure to AZB was associated with increases in relative and absolute peripheral CD3^+^, CD4^+^, and CD8^+^ cell counts (60). When used as a supplementary therapy in children with solid malignancies, inclusion of AZB in combination chemotherapy regimens contributed to significant reductions in the frequency of infectious bacterial and viral complications in addition to improvements in immunological Markers (71). In children with Hodgkin’s lymphoma and histiocytosis, administration of AZB was associated with increased T-cell counts, neutrophil activity and bacterial activity, activation of humoral immunity, and decreases in tumour mass of 20–30% (66). In a study of treatment in patients with chronic lymphocytic leukaemia, the addition of AZB to cyclophosphamide and chlorbutin in patients with stage III B-cell lymphocytic leukaemia saw positive changes in CD3^+^ and CD4^+^ T-cells, natural killer (NK) cell counts, increased phagocytic activity of neutrophils and immunoglobulin levels over time were also noted. AZB contributed to increased chemotherapy efficacy, as well as anti-bacterial therapy in the development of infectious and inflammatory complications. The clinical and immunological effects associated with AZB administration were maintained for 140 days (65).

**Table 1:** Oncology Studies with Azoximer bromide.

Nosology	Scheme/dose	Subjects	Results
**Colon cancer, III-IV stages**	Adjuvant regimen, Double-blind placebo-controlled study Group 1; 6 mg + 5 FU, Group 2; 12 mg + 5 FU, Group 3; 12 mg + 5 FU	49	Positive changes in immunological indicators over time (87.2% of treatment patients in all experimental groups). Significant increase of relative and absolute white blood cell (CD3, CD4, CD8) count (60).
**Breast cancer**			
Nodular breast cancer (stage T1- _2_N_0_M_0_)	AZB monotherapy, 6 mg every other day (10 injections)	96	Normalization of immune system’s humoral components and cellular- mediated indicators (61).
Underwent FAC chemotherapy + some radiation therapy	Adjuvant regimen, 6 mg (9 injections between chemotherapy courses)	94	Improving chemotherapy tolerance (according to NCI-CTC V3.0), and quality of life (FACT-G questionnaires), absence of adverse events. Increased activation of immunity indicators (CD50, CD38, CD95 and CD11b) on blood lymphocytes. Absolute increase in the number of immunity parameters (mature B-cells, CD 45 RA+ cells, T-helper cells). Stimulating leukopoiesis due to increase of pro-inflammatory induction (62).
Underwent core-biopsy	Neoadjuvant regimen, 12 mg every other day (5 injections)	20	Absence of complications and side effects. Therapeutic pathomorphosis of varying degree and changes in the composition of intratumoral lymphocytes (in 30% of cases) (63, 64).
Primary operable breast cancer	Neoadjuvant regimen, 12 mg every other day (5 injections)	75	Therapeutic pathomorphosis of varying degree in the primary tumor and metastasic lymph nodes (63).
Chronic lymphocytic leukemia	CT + AZB or AZB monotherapy	21	Increased T-cell counts and phagocytic activity of neutrophils and immunoglobulin levels over time. Increased the efficiency of standard chemotherapy, and increased antibacterial therapy in the development of infectious and inflammatory complications (65).
Hodgkin’s lymphoma	CT + AZB; 6 mg (children >5 years; 3 mg), for 2 weeks 3x per week, 2 courses	16	Decreased T-killer counts, partial normalization of immunoregulatory cells ratio, activation of humoral immunity indicators, and increased phagocytic activity. Significant reduction in the size of tumor peripheral lymph nodes by 30–60% (66).
Kidney cancer	AZB monotherapy, adjuvant regimen	72	Increased immunoregulatory cells ratio and normalization of immunity indicators (67).
Langerhans cell histiocytosis	CT + AZB; 6 mg (children >5 years; 3 mg), for 2 weeks 3x per week, then for 6 weeks 2x per week	12	Increased T-cell counts, neutrophil and bactericidal activity. Activation of humoral immunity. Normalization of immunity indicators, Significant reduction in the size of tumor volume by 20–30% (66).
Lung cancer			
Advanced lung cancer	CT + AZB	129	Significant increase of overall survival rate (p=0.0164) and reduced frequency and severity of infectious complications during chemotherapy (68).
Localized lung cancer	AZB monotherapy	56	Normalization of immunity indicators, and significant reduction of metastatic frequency (p=0.017). Sings of activation of specific T-cells and non-specific cell mediated immunity (67).
Skin melanoma	AZB monotherapy, adjuvant regimen 6–12 mg every other day for 3 months	40	High drug efficacy and increase of 3-year survival rate of patients (92.5%). Decrease in the frequency of cytogenetic disorders and the probability of disease relapsing (47.5%) (69, 70).
Solid tumors in children (ES, NB, rhabdomyosarcoma, germ cell tumors)	CT + AZB subcutaneously 0.15 mg for 5 days	20	Significant decrease in the incidence of incidence of infectious bacterial (1.8%) and viral (12.5%) complications (p<0.05). Reduction of immunosuppression severity and significant improvement of immunity indicators (p<0.05) (71).

Several studies have investigated the potential of AZB in the lung cancer setting, particularly advanced lung cancer. In one study, the addition of AZB to standard chemotherapy resulted in a significant increase in overall patient survival (p=0.0164). These patients also had a lower number of chemotherapy complications, compared to untreated patients and a subgroup of patients where AZB was used only during the final courses of chemotherapy (68). In patients with localised lung cancer (stage I-II), adjuvant AZB monotherapy following surgical treatment resulted in a significant reduction in the reported frequency of metastasis. In addition, patients in the AZB group showed signs of activation of specific T-cell and non-specific cell-mediated (neutrophilic phagocytes and NK cells) immunity (67).

Two studies have assessed the effect of AZB on the tolerability of postoperative chemotherapy or chemo-radiotherapy in patients with breast cancer. The first study included 94 patients with breast cancer after radical surgical treatment who received fluorouracil, adriamycin, and cytoxan (FAC) combination chemotherapy and some radiation therapy. The study showed favourable tolerability of AZB with the absence of any adverse events. Treatment saw increases in activation antigens such as ICAM-3 (CD50), CD38, CD95, and CD11b on blood lymphocytes, compared with control. Treatment also saw a marked reduction in the chemotherapy-related toxicity in the form of decreased levels of neutropenia and fewer infectious complications in the AZB group (62).

The effect of AZB on the tolerance to postoperative chemotherapy or chemo-radiotherapy has been investigated in patients with breast cancer. Administration of AZB in the intervals between FAC courses improved treatment tolerability and significantly reduced the incidence of adverse effects. The associated changes in peripheral blood cell count changes and immunological parameters indicated a positive immunomodulatory effect of AZB, particularly in patients with reduced cell-mediated immunity at baseline (62). In a further study of patients with nodular breast cancer in the early stages of neoplasia (stage T_1_–_2_N_0_M_0_), AZB was associated with greater normalisation of humoral immunity indicators (Ig G and Ig M) than standard therapy. The authors concluded that use of AZB in traditional complex therapy of breast cancer at the initial stages of the disease provides a pronounced positive change of the immune status in the form of normalisation of not only cell-mediated indicators but also the humoral component of the immune system (61).

The potential benefits of preoperative AZB use was investigated, with focus placed on the tumour microenvironment. Tumour core biopsies were collected from 20 patients with breast cancer prior to and after AZB treatment. Immunohistochemical findings implied that AZB had an immunomodulatory effect on intra-tumour cells. Decreased CD4^+^ cell counts and T-cytotoxic (CD8^+^) lymphocyte counts were observed where initial levels were above the median, and increased counts were seen where initial levels were below the median (median for CD8^+^ cell count is 41%, for CD4^+^ cell count is 47%). In addition, AZB appeared to have a positive effect on the tumour itself (in the form of pathomorphosis induction), associated with a subpopulation of intra-tumoural CD4^+^ lymphocytes and decreased CD8^+^/CD4^+^ index. According to histological examination of surgically removed tumour tissue 7 days following AZB immunotherapy, 30% of patients had responded and one patient had a complete pathological response (grade IV; triple-negative breast cancer) (63, 65).

The effect was investigated further in a larger study conducted in primary operable breast cancer (63, 64). Patients underwent a radical mastectomy 8 days after AZB dosing, with the removed tissue undergoing histological examination and determination of the degree of tumour therapeutic pathomorphosis according to the Lavnikova scale (72). Evaluation of the primary tumour indicated therapeutic pathomorphosis of various degrees in 64% of the patients, with grade I pathomorphosis being noted in most cases (58%). Pathomorphosis in metastatic lymph nodes was observed less frequently (22.7%) than in the primary tumour. In all cases, pathomorphosis severity of metastatic tumour cells in the lymph nodes was lower than in the primary tumour (grade I). Tissue pathomorphosis was not observed in patients that did not receive AZB (63).

A well-marked anticancer effect of AZB administered alone as adjuvant therapy was also observed in patients with skin melanoma (morphologically verified stages I–III). Cytogenetic examination revealed 8–40% of lymphocytes with various cytogenetic disorders in all 70 patients (69, 70). Patients with skin carcinoma who received AZB immunotherapy after surgical intervention (3-month courses of 6–12 mg), showed a 92.5% 3-year survival rate, compared to 13.3% who received surgical treatment only. In addition, AZB treatment was correlated with a lower probability (47.5%) of disease relapsing *versus* 93.3% of the control group (69, 70).

## Conclusion

This literature review details the history of a molecule that has received little attention in the traditional, English-speaking journals. Developed in the 1970s and 80s, AZB is widely used in Russia and Slovakia and estimates suggest that it has been used as an adjuvant in over 400 million vaccine doses.

Clinical observations and research papers on the use of AZB in cancer patients together with a favourable safety and tolerability profile allows us to consider AZB as a promising anticancer agent for use both as part of complex/combination therapy as well as monotherapy.

## Author Contributions

LG, NF, and NT contributed to conception and design of the manuscript, literature review of previous related articles, and wrote sections of the manuscript. All authors contributed to the article and approved the submitted version.

## Funding

All sources of funding were provided provided in the form of an educational grant from Petrovax NPO.

## Conflict of Interest

The authors declare that the research was conducted in the absence of any commercial or financial relationships that could be construed as a potential conflict of interest.

## Publisher’s Note

All claims expressed in this article are solely those of the authors and do not necessarily represent those of their affiliated organizations, or those of the publisher, the editors and the reviewers. Any product that may be evaluated in this article, or claim that may be made by its manufacturer, is not guaranteed or endorsed by the publisher.
